# Ventricular fibrillation during carotid endarterectomy and bailout stenting: a case report

**DOI:** 10.1186/s40981-022-00517-4

**Published:** 2022-03-31

**Authors:** Yuji Ueda, Masaaki Asamoto, Taro Kariya, Takafumi Akai, Katsuyuki Hoshina, Kanji Uchida

**Affiliations:** 1 Department of Anesthesiology and Pain Relief Center, The University of Tokyo Hospital, 7-3-1, Hongo, Bunkyo-ku, Tokyo, 113-8655 Japan; 2 Department of Vascular Surgery, The University of Tokyo Hospital, 7-3-1, Hongo, Bunkyo-ku, Tokyo, 113-8655 Japan

**Keywords:** Ventricular fibrillation, QT prolongation, QT dispersion, Carotid Endarterectomy, Fatal arrhythmias, Hypercapnia, Hypomagnesemia, Intraoperative, Perioperative, Carotid artery manipulation

## Abstract

**Background:**

Carotid artery manipulation is not a special technique but reports of intraoperative ventricular fibrillation are rare. The risk of fatal arrhythmias may be hidden behind routine surgical techniques and anesthetic management. We focused on QT prolongation and QT dispersion.

**Case presentation:**

A 77-year-old man underwent carotid endarterectomy and bailout stenting. Although there were no obvious preoperative risk factors for intraoperative ventricular tachyarrhythmia, ventricular fibrillation (VF) had occurred during a maneuver of the carotid artery under hypercapnia. QTc was prolonged from 317 ms before surgery to 458 ms before the onset of VF. QTc dispersion between leads II and III was also increased to 50 ms. Hypomagnesemia was noted after resuscitation by electrical defibrillation, adrenaline, and noradrenaline.

**Conclusions:**

We considered that the combination of multiple risk factors led to the development of ventricular fibrillation. It should be noted that carotid artery manipulation has the potential to cause arrhythmias.

## Background

Fatal tachyarrhythmias during noncardiac surgery are rare and are associated with diverse factors, including intraoperative hemorrhage, cardiac disease, drugs, electrolyte abnormalities, and surgical techniques. Fatal tachyarrhythmias, including ventricular fibrillation (VF) during carotid endarterectomy (CEA), have not been reported to date and are believed to occur rarely [1]. Here, we report a 77-year-old male patient with sudden VF during CEA. We investigated the cause of this rare event.

The case was prepared according to CARE guidelines.

## Case presentation

A 77-years-old, male (height, 163 cm; weight, 52 kg) was scheduled for carotid endarterectomy (CEA) due to right internal carotid artery stenosis. He had a history of atrial fibrillation for 8 years, spinal canal stenosis, arteriosclerosis obliterans, diabetes mellitus, hypertension, dyslipidemia, right lower extremity ulceration due to diabetes mellitus, and chronic kidney disease without hemodialysis. He took 0.625 mg/day bisoprolol fumarate, 20 mg/day nifedipine, and 30 mg/day edoxaban tosilate hydrate. Preoperative electrocardiogram (ECG) showed atrial fibrillation with a heart rate of 47/min, complete right bundle branch block, and inverted T waves in V1–4 leads with a normal QRS axis. Echocardiography showed an ejection fraction (EF) of 66%, no wall motion abnormalities, and no valvular disease. Blood tests showed blood urea nitrogen (BUN) 31.8 mg/dl, creatinine 2.46 mg/dl, Na 138 mmol/L, K 5.3 mmol/L, and Mg 2.1 mg/dL. HbA1c is 5.9%. His exercise tolerance exceeded four metabolic equivalents (METs).

General anesthesia was induced with propofol and fentanyl, and rocuronium was administered to facilitate tracheal intubation. Remifentanil, fentanyl, and desflurane were administered to maintain general anesthesia. After induction of anesthesia, atrial fibrillation returned to sinus rhythm. Upon the surgeon’s request, continuous intravenous phenylephrine was initiated to maintain systolic blood pressure in the range of 120 to 160 mmHg. A near-infrared brain oxygen monitor (NIRO) was applied to both sides of the forehead. After clamping the right carotid artery, the tissue oxygenation index (TOI) from the NIRO gradually decreased from 53 to 27%. Due to the patient’s hyperbranched right carotid artery, it was difficult for the surgeon to closely check the peripheral anastomosis site. During surgery, bleeding from the needle hole occurred. Severe calcification of the carotid artery prevented the detection of bleeding, and short-term bleeding reached up to 1600 ml. Even after completion of hemostasis, a low TOI persisted (19%). Intraoperative carotid angiography revealed carotid artery dissection, and a bailout stenting was planned. To maintain cerebral blood flow thereafter, hypercapnic ventilation (Max PaCO2 59.5 mmHg) was employed at this point. The TOI improved to 66%.

**Fig. 1:**
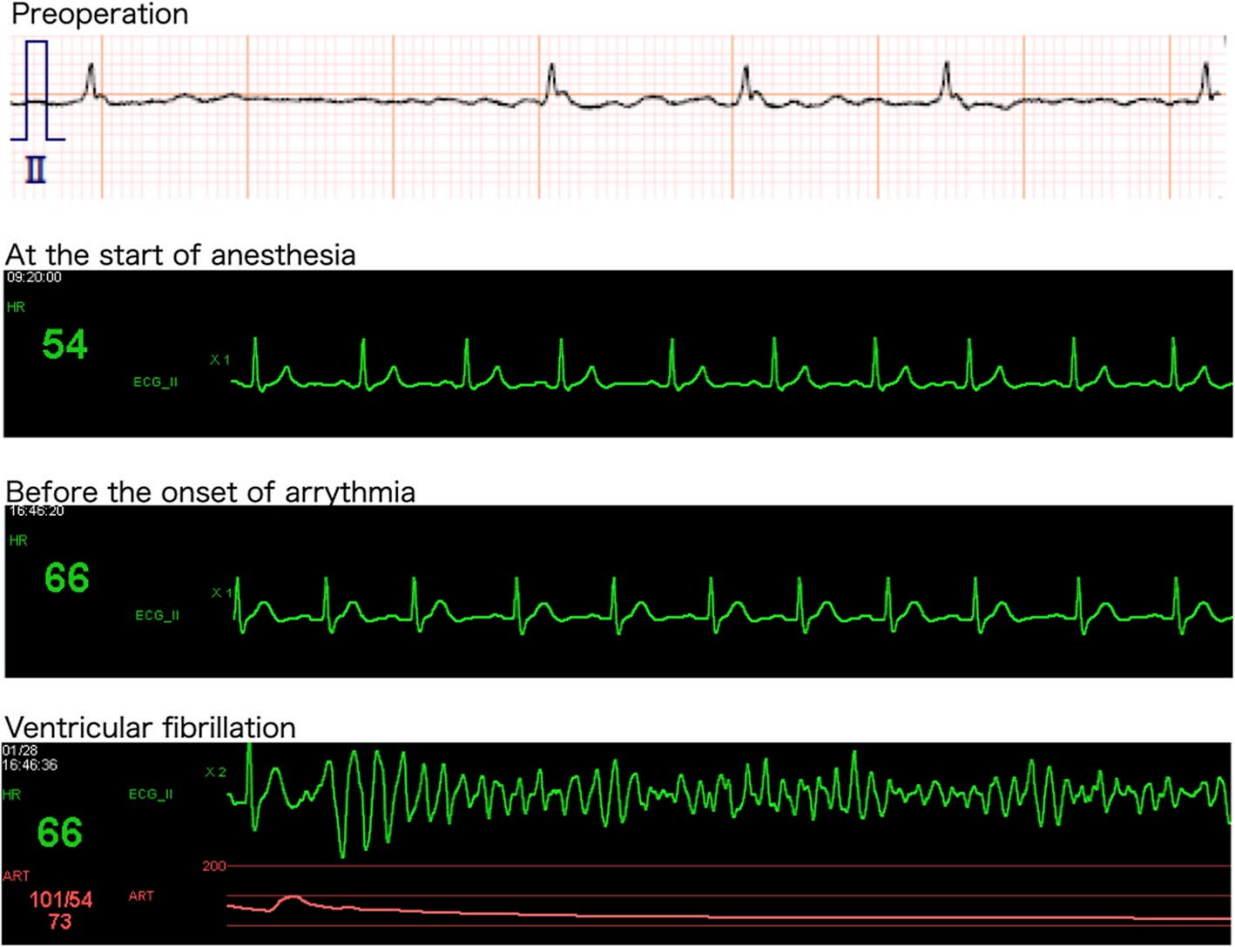
Electrocardiogram lead II. Atrial fibrillation converted to sinus rhythm after induction of general anesthesia and during surgery. Ventricular fibrillation developed when the surgeon touched the carotid artery

When the surgeon touched the dissected carotid artery before the guidewire crossed the true lumen of the lesion, VF had occurred (Fig. 1). The anesthesiologist immediately recognized the VF and alarmed surgeons to stop surgery and start chest compressions.

Electrical defibrillation (biphasic 150 J) was performed 1 min later, which quickly restored sinus rhythm. After the sinus rhythm had been restored, systemic hypotension persisted, requiring incremental intravenous bolus noradrenaline (0.1 mg), and chest compressions were required. After 0.3 mg of intravenous adrenaline was administered three minutes after the onset of VF, blood pressure recovered to the normal range. No blood electrolyte abnormalities other than hypomagnesemia (Na 134 mmol/L, K 4.6 mmol/L, Cl 109 mmol/L, Ca^2+^ 1.09 mmol/L, Mg 1.6 mg/dL) were found. ECG showed inverted T waves in V1–4 leads, flat T waves in V5–6, and QT prolongation. Blood transfusions and continuous noradrenaline and landiolol infusions were initiated. The surgical procedure was resumed after the hemodynamics stabilized. The duration of surgery was 7 h 21 min, and the duration of anesthesia was 9 h 58 min.

The patient was transferred to the ICU postoperatively with the trachea intubated in a stable condition. On ICU admission, V4–6 ST depression was noted, and echocardiography showed hypokinesis in the anterior to the apex and inferior left ventricular wall. A high-sensitivity cardiac troponin I (cTnl) level was elevated (6335pg/ml, on POD1). Although reduced wall motion and elevated cardiac enzyme levels led us to suspect myocardial ischemia, coronary angiography was avoided due to the possibility of further deterioration of renal function with the use of contrast media. The patient was extubated on postoperative day (POD) 1. The catecholamine support was weaned off on POD 2. He was discharged from the ICU and moved to the general ward on postoperative day (POD) 6. There was no further recurrence of life-threatening ventricular arrhythmia. The patient was discharged on postoperative day 18 without further examination. Three months after discharge, the patient was readmitted to the hospital for gastrointestinal bleeding. During hospitalization, the patient died of intractable and progressive heart failure.

## Discussion

We experienced a patient with VF during the manipulation of a guidewire for bailout carotid stenting during CEA. There were no obvious preoperative risk factors for intraoperative ventricular tachyarrhythmia. No remarkable family or medication history was noted. The results of preoperative blood testing, standard 12-lead ECG, and transthoracic echocardiography were all negative for potential arrhythmia. In particular, the preoperative QT interval was within the normal range. The preoperative QTc was 317 ms, as measured using the tangent method suggested for use in atrial fibrillation [2].

The QT interval is one of the quantitative values associated with the prevalence of ventricular tachyarrhythmia. Reportedly, approximately 80% of patients had a longer QTc after surgery under general anesthesia than those before surgery [3]. In our case, the QTc had increased to 411 ms at the time of anesthesia induction and further increased to 458 ms before the onset of arrhythmia. Each 10-ms increase in QTc contributes approximately a 5% to 7% exponential increase in the risk of TdP in congenital LQTS patients [2]. We concluded that there was significant intraoperative QT prolongation in this case.

**Fig. 2:**
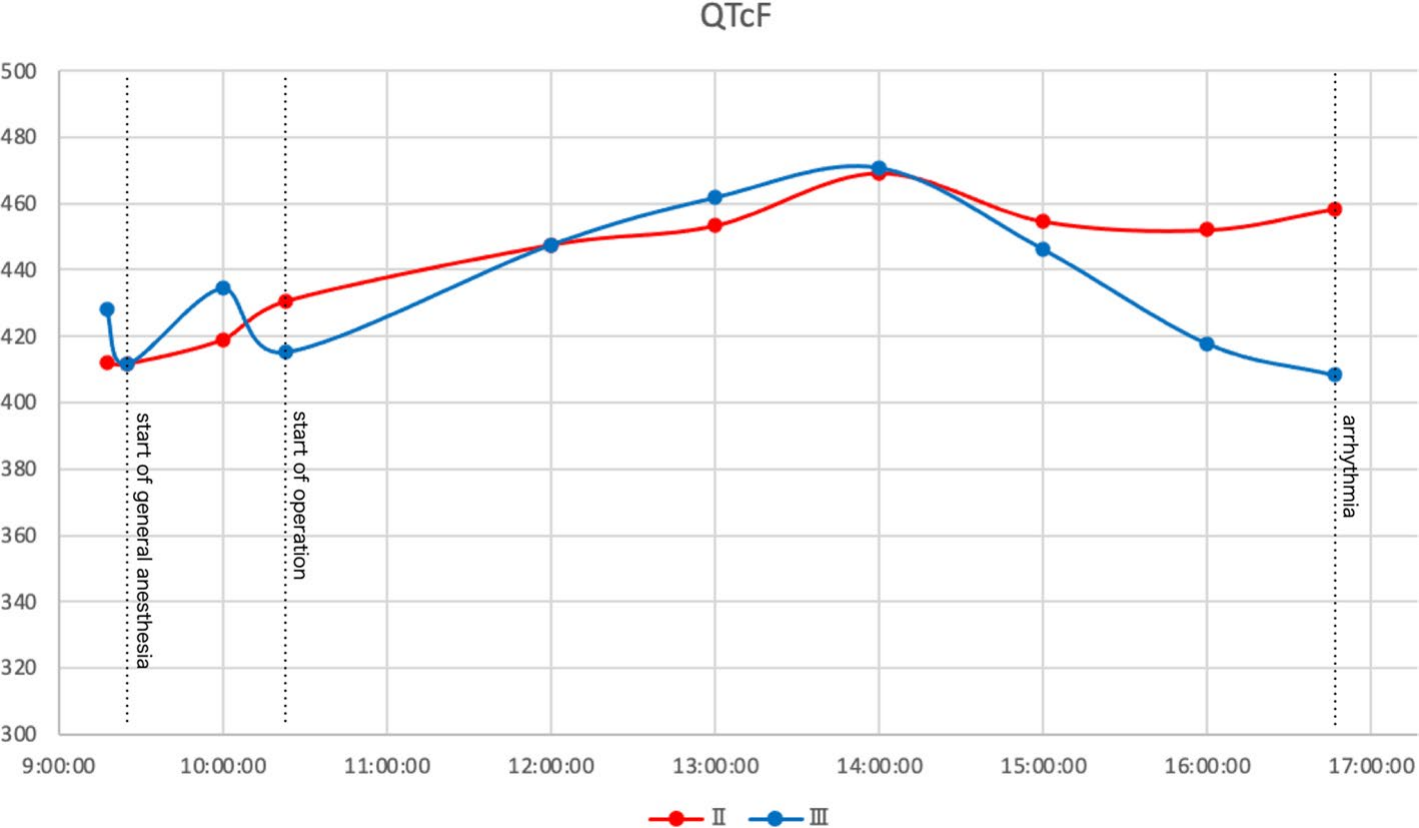
Time course of QTc of leads II and III. The difference of QTcF between leads II and III increased in the latter half of the recording and reached 50 ms of difference just before the onset of the ventricular tachyarrhythmia

It is widely known that different types of cardiomyocytes have different electrophysiological characteristics and are distributed heterogeneously [4]. The spatial heterogeneity of myocardial depolarization results in QT dispersion. Increased QT dispersion seems to be associated with ventricular tachyarrhythmia [5]. At our institution, intraoperative waveform data are stored only for leads II and III. In general, QT dispersion is defined as the difference between the maximum and minimum QT values in a 12-lead ECG [6]. In this study, general QT dispersion was not available, but we were able to measure QTc in leads II and III (Fig. 2). Initially, there was no difference between the two leads, but the QTc difference increased intraoperatively and reached 50 ms just before the onset of ventricular tachyarrhythmia.

We anesthetized the patient with hypercapnia for 4 h. This was to prevent cerebral ischemia. On the other hand, hypercapnia increases both QTc interval and QT dispersion [7]. Permissive hypercapnia may provide an environment for arrhythmogenesis.

Hypomagnesemia is an important risk factor for arrhythmias [8]. Although the mechanism is not clear, magnesium is believed to equalize repolarization. Furthermore, some reports have found a correlation between Mg and QT dispersion [9]. In the present case, hypomagnesemia was observed on postoperative examination.

In this case, there were no circulatory changes before the onset of VF.

Myocardial infarction was denied because cardiac function recovered quickly.

Takotsubo cardiomyopathy is another candidate because it can prolong QT and cause TdP [10]. The suggested mechanism of QT elongation in Takotsubo cardiomyopathy is catecholamine or autonomic effects on the myocardium. However, postoperative echocardiography in the ICU did not reveal any obvious findings suggesting takotsubo cardiomyopathy.

We used phenylephrine up to 0.5 μg-kg-1-min-1 to maintain blood pressure. Some studies have found no QT prolongation or QT dispersion with phenylephrine [11], while other studies on the relationship between afterload and QT dispersion have shown that phenylephrine causes QT dispersion [12].

The possibility of structural changes in the ventricular muscle predisposing ventricular tachyarrhythmia should also be considered. Aging is a well-known risk factor for the development of arrhythmias [13]. In the present study, factors that increase myocardial remodeling could be related to old age, hypertension, diabetes mellitus, and atrial fibrillation. Abnormalities in the myocardial gap junctions and fibrosis of the tissue interstitium seem to lead to the development of heterogeneous repolarization [14].

Finally, the surgical procedure could trigger VF. The ECG trace showed no signs of dysrhythmia before VF onset, and there is no doubt that carotid artery manipulation was the trigger.

There are also reports of TdP developing from carotid dissection [15] and ventricular fibrillation occurring after carotid sinus massage [16]. Moreover, although there are no reports of VF occurring during CEA, we should be aware that carotid manipulation may cause sudden onset of lethal ventricular tachyarrhythmias.

CEA causes the balance of the cardiac autonomic nervous system to be biased toward the sympathetic side [17].

The relative increase in sympathetic modulation after CEA is likely mediated by alterations in the sensitivity of carotid sinus baroreceptors [18].

In conclusion, QT prolongation, QT dispersion, hypercapnia, hypomagnesemia, acute heart disease, use of phenylephrine, advanced age, and carotid artery manipulation were considered as potential causes of intraoperative VF.

## Data Availability

Not applicable.

## Abbreviations

BUN: Blood urea nitrogen
CEA: Carotid endarterectomy
CTnI: Cardiac troponin I
ECG: Electrocardiogram
EF: Ejection fraction
MET: Metabolic equivalents
NIRO: Near-infrared brain oxygen monitor
POD: Postoperative day
QTc: Corrected QT interval
QTcF: Corrected QT interval; Fridericia’s formula
TdP: Torsades de Pointes
TOI: Tissue oxygenation index
VF: Ventricular fibrillation
